# Vaccine nationalism will persist: global public goods need effective engagement of global citizens

**DOI:** 10.1186/s12992-022-00802-y

**Published:** 2022-02-12

**Authors:** Stuart J. Peacock

**Affiliations:** 1 Faculty of Health Sciences, Simon Fraser University, Burnaby, BC Canada; 2 Cancer Control Research, BC Cancer, Vancouver, BC Canada; 3 Canadian Centre for Applied Research in Cancer Control, Vancouver, BC Canada

**Keywords:** Covid-19, Vaccines, Vaccine nationalism, Global public goods, Governance, Citizen engagement, Public engagement

## Abstract

Covid-19 presents a unique opportunity to transform democratic engagement in the governance of global public goods. In this paper, I describe a global public goods framework and how it relates to Covid-19 vaccines, and summarize some of the global responses to Covid-19. I discuss some of the global threats to health and prosperity posed by the inequitable distribution of vaccines, and propose transformative thinking to democratically engage citizens in the governance of global public goods. In recent years, public-private partnerships and philanthropic organizations have successfully stepped in to help international organizations like the UN and WHO provide global public goods, but they are not democratically elected or publicly accountable. Global public goods are critical to addressing Covid-19, future pandemic preparedness, global health policy, health equity, and the unfolding climate crisis. To make us more resistant and resilient to future global health crises we need transformative thinking to democratically engage global citizens. We need to lay the foundations for a ‘global social contract’ on global public goods.

## Background

Covid-19 has demonstrated that national governments are ill-equipped to deal with truly global health crises, and highlighted the importance of global public goods. ‘Vaccine nationalism’ has exposed deep fault lines in an increasingly globalized world, pitting high-income nations against each other, and against middle- and low-income countries. In particular, recent UK and European disputes relating to the production and distribution of Covid-19 vaccines have shown how high-income nations may employ inwardly focused vaccine policies that pose threats to the health and prosperity of other countries. At the same time, the global response to Covid-19 has shown how international cooperation can help improve the equitable distribution of vaccines to all countries, not just the wealthy. National and international responses to Covid-19 point to the need to develop more effective frameworks for the governance of global public goods, frameworks which must include international institutions such as the UN and WHO, nation-states, as well as public-private and philanthropic organizations.

This article is organized as follows. First, I briefly describe the framework of global public goods and how it relates to Covid-19 vaccines. Second, I summarize some of the global responses to Covid-19, including the UK and EU dispute that framed this paper. Third, I discuss some of the global threats to health and prosperity posed by the inequitable distribution of vaccines. Finally, I propose transformative thinking to democratically engage citizens in the governance of global public goods.

## Covid-19 vaccines and global public goods

Over the last year, there have been multiple calls from the WHO, UN and other international organizations for effective vaccination against Covid-19 to be declared a *global public good:* vaccination should made be *non-rival* and *non-excludable* so everyone can benefit.

Private ‘goods’ possess two key features: they are *rival* and *excludable* [1]. The definition of *rival* is that one person’s consumption of a particular good reduces another person’s ability to consume the same good. The definition of *excludable* relates to whether people can be prevented from consuming a given good [1]. Examples of private goods include food, clothes, mobile phones, or services like travel. There are usually limited quantities of these goods, and owners and sellers can prevent other individuals from enjoying their benefits. Most goods are private because their consumption can be withheld until a payment is made in exchange for them, and once consumed they cannot be consumed again [2].

Public ‘goods’ also have two key features: they are *non-rival* and *non-excludable*. The definition of *non-rival* is that people can consume the product without reducing the amount available of that product for anyone else. The definition of *non-excludable* is that once a product in provided or produced it is impossible to prevent people who have not paid from it from being able to consume it [1]. Examples of public goods include national security, clean drinking water, clean air that we breathe, as well as public parks and street lighting.

*Global public goods* can be thought of as public goods which provide benefits that cross borders and are available to the global population [2, 3]. The eradication of a disease, such as smallpox, provides an example of a global public good: once smallpox was eradicated the whole of humanity benefited irrespective of the country they lived in. Other examples of global public goods include clean air, biodiversity, international security, and economic prosperity. What makes goods private or public is not how they are produced but instead relates to whether the good provides wider benefits to the public. The eradication of disease can be thought as a global public good, and vaccines are one element in providing that good. But, the vaccines themselves can be made both rival and excludable, ensuring they are private goods which can be traded in markets. Alternatively, governments can make vaccines public goods through policy choices and legislation.

Although there are significant benefits from global public goods, they are typically under-produced and inequitably allocated because there is no commercial incentive to provide them [2]. Global public goods do not command a price in the market, as a result private sector companies cannot recoup the cost of their production. In the case of national public goods, the government of the nation-state will often step in to provide the public good directly, or create incentives for its production through taxation, licenses etc. In the case of global public goods, there is no single ‘global government’ to address the challenge. The issue then becomes how to best create and sustain international collaborative action that ensures the production of global public goods worldwide.

In the context of Covid-19, much of the funding and scientific research on which the development of Covid-19 vaccines has been based has come from governments. The majority of the pioneering work on mRNA vaccines was carried out using public research funds (much of it from the US National Institutes of Health), but private sector manufacturers stand to make large profits [4]. A recent UK paper has suggested that 97–99% of the funding for the Oxford-AstraZeneca Covid-19 vaccine came from public sources, including governments and philanthropic organizations [5]. However, pharmaceutical companies have a strong incentive to ensure that vaccine doses are made rival and excludable. By retaining property rights over Covid-19 vaccines, companies can control supply and influence the price at which they are sold. Limiting the supply of vaccines may drive prices up at the expense of poorer countries who cannot afford them. Pharmaceutical companies also have a strong incentive not to share intellectual property relating to their vaccine because they may wish to develop that knowledge further, and use it to develop other therapeutics (such as treatments for cancer, HIV etc.). Some commentators have gone further, suggesting that prolonging the pandemic provides a strong incentive for the private companies to limit vaccine supply, leaving a substantial pool of unvaccinated populations, which would then driving the need for boosters or modified vaccines [6].

In June 2021, the UN Secretary General said he “was encouraged by the announcement made ahead of the G7, by the International Monetary Fund (IMF) together with the World Bank, WHO and World Trade Organization (WTO), regarding a $50 billion programme to support vaccination delivery in developing countries” as part of his call for Covid-19 vaccination to be considered a global public good [7]. At the 2020 World Health Assembly, countries supported the following UN resolution text on global public goods: “the role of extensive immunisation against COVID-19 as a global public good for health in preventing, containing and stopping transmission in order to bring the pandemic to an end, once safe, quality, efficacious, effective, accessible and affordable vaccines are available …” [8]. UNESCO’s International Bioethics Committee (IBC) and the World Commission on the Ethics of Scientific Knowledge and Technology (COMEST) have also called for vaccines to be treated as a global public good [9]. They called on the IMF to allow for lower-income countries to draw on Special Drawing Rights to fund the development of vaccines, while higher-income countries should continue to fund WHO Covid-19 vaccines initiatives. They also called on pharmaceutical companies to share intellectual property to enable the manufacturing of vaccines in lower-income settings, and to support vaccines for all. Some academics have suggested that a Global Fund for Public Goods (GFPG) should be created to ensure sustainable financing for global public goods [10]. This model would require all countries to make funding contributions for research, development and manufacture of Covid-19 vaccines to help reduce barriers to making vaccination a global public good.

## The global response to Covid-19

The Access to Covid-19 Tools (ACT) Accelerator was launched by the WHO and partners in April 2020, with the aims of accelerating the development, production and equitable access to Covid-19 tests, treatments, and vaccines. While the ACT Accelerator was designed to be a global leader in the response to Covid-19, it has only been partly successful in doing so. Several countries, including the US, have sought to develop their own, unilaterally based, global response to Covid-19. COVAX is a key component of the ACT Accelerator, co-convened by the Coalition for Epidemic Preparedness Innovations (CEPI), GAVI (the Vaccine Alliance) and the WHO. It works in partnership with UNICEF, vaccine manufacturers, the World Bank, and philanthropic foundations. COVAX is the only global initiative that is working with governments and manufacturers to ensure Covid-19 vaccines are available worldwide, and specifically to middle-, and low-income countries. COVAX has recently stated its goal is to deliver 2 billion vaccine doses in 2021, and 1.8 billion to 92 lower income economies [11]. COVAX allocates Covid-19 vaccines, GAVI guarantees purchases of predetermined numbers of doses to encourage the private sector to develop and manufacture new vaccines, while other pillars of the ACT Accelerator focus on Covid-19 diagnostics and therapeutics [12]. The WHO estimate they need USD $38·1 billion in funding, but despite large pledges from governments, the private sector and philanthropic organizations, as of July 23, 2021 there is still a shortfall of USD $16.6 billion [12]. At their recent summit, G7 countries committed to share an additional 870 million doses of vaccines to support equitable access for 2021–22, taking G7’s total commitment since February 2021 to one billion doses [13].

That said, some commentators have raised significant questions about COVAX [14]. COVAX is highly reliant on a wide range of stakeholders, including both governments and the private sector. COVAX relies on manufacturers to donate vaccine doses, but it has struggled to secure both agreements with manufacturers, and the physical vaccine doses themselves [14]. While the US recently donated 500 million doses to COVAX, this was achieved by diverting dollars away from existing vaccination drives in lower income countries [14]. This may mean cuts to funding for training people to administer vaccines, or to fuel for transport. COVAX faces a large power imbalance, with high-income countries and pharmaceutical companies holding most influence. Pharmaceutical companies have been resistant to making large donations of vaccine doses. And, bureaucratic barriers have delayed the disbursement of over $200 million to vaccination programs in lower income countries [14].

The WHO has also established the Covid-19 Technology Access Pool (C-TAP) to facilitate timely, equitable and affordable access to Covid-19 health products by boosting their supply. In June 2021, the WHO announced that it is supporting a South African consortium to establish the first Covid-19 mRNA technology transfer hub. The facility will allow manufacturers from middle- and low-income countries to receive training in how to produce vaccines, quality control, and the relevant licenses to do so, in order to scale-up access to vaccines and facilitate broad and rapid technology transfer to multiple recipients. C-TAP still faces significant barriers to the effective transfer of Covid-19 technologies, especially as many countries do not have measures in place to ensure that pharmaceutical countries engage in technology sharing initiatives [15].

In spite of these global efforts, equitable access to Covid-19 vaccines remains a divisive international issue. Worldwide only 13.51% of the population have been fully vaccinated, compared to 45.44% in the EU, and 54.15% in the UK [16]. In particular, recent European disputes relating to the production and distribution of Covid-19 vaccines have highlighted how some high-income nations are employing nationally focussed vaccine policies that pose threats to the health and prosperity of other countries.

### UK vaccine policy and splendid isolation

Despite the strong global response to Covid-19, some nations have adopted policies of ‘vaccine nationalism’ or ‘my country first’ [17]. These policies will blunt some of the international efforts to allocate vaccines rapidly and equitably. Some of the actions taken by the UK Government during the pandemic can be viewed as examples of ‘vaccine nationalism’. However, in this paper I would like to suggest that the UK Government’s actions on vaccines should also be conceptualized in terms of long-standing British policies based on notions of ‘splendid isolation’. The term ‘splendid isolation’ was introduced by Prime Minister Lord Salisbury in a speech in 1898, and it has been a mainstay in British policy documents ever since [18]. Specifically, Salisbury was concerned that British foreign policy should be built on freedom from international intrigues and entanglements [19]. In popular rhetoric, splendid isolation builds on recurring nationalistic themes of Britain as an island nation, protecting itself (in as far as it can) from European conflicts, diseases and epidemics. The rhetoric of Britain in ‘splendid isolation’ as an island nation was prominent in the recent Brexit campaign, specifically in relation to anti-migration rhetoric about freedom of movement under EU rules. It is important to note that the UK plays an active role in many international agencies, such as the UN, WHO, the World Bank, and up until recently the EU. And, the UK is playing a role in global efforts to allocate Covid-19 vaccines equitably. What I am suggesting is that some (but not all) of the UK Government’s actions on Covid-19 vaccines should be viewed as policies based on notions of ‘splendid isolation’.

Early in 2020, the UK was concerned that Pfizer and Moderna would favour their home USA market (under pressure from the Trump Administration), prompting the creation of the UK Vaccine Taskforce [20]. While the UK experienced late lockdowns, confused test and trace roll-outs, and soaring mortality rates, its vaccine program is the envy of many countries. The UK Taskforce, working with a strong public-private coalition, allowed the country to act early and decisively, reenergizing vaccine manufacturing, and scaling up production quickly. Arguably, the UK’s vaccine policy of splendid isolation served it well. The UK has reinvigorated domestic vaccine manufacturing, and signed deals with seven manufacturers for over 400 million doses. By February 2021, the government had vaccinated long-term care residents, people over 70, and many front line health workers. Infection and mortality rates are falling, and by July 54.15% of the population were fully vaccinated [16].

However, a recent paper has suggested that 97–99% of the funding for the Oxford-AstraZeneca Covid-19 vaccine came from public sources, built on 20 years of existing research and development. The authors suggest there is a severe lack of transparency in reporting of research funding mechanisms for Covid-19 vaccines [5]. In August 2021, the Financial Times reported that the UK government was set to recommend booster doses of Covid-19 vaccines for up to 500,000 immuno-compromised individuals, as well as for all people over 70 years [21]. The roll out of boosters commenced in September 2021, despite protests from the WHO Director General, who warned that vaccine nationalism increased the risk of new variants emerging. The WHO has called on high-income countries to share doses allocated for boosters with lower-income nations, and for a moratorium on all booster doses until the end of 2021. The UK has largely ignored these calls.

While the UK government has a moral argument for prioritizing vaccines for its citizens; its moral responsibilities do not end at its shores. The UK’s commitments to COVAX demonstrates it is concerned about alleviating suffering in middle- and low-income countries, but the government has been clear that its citizens come first.

### Covid-19 vaccine trade bans will achieve little

On January 25th 2021, the EU threatened to block exports of Covid-19 vaccines to non EU-bloc countries, with concerns that AstraZeneca was about to fall short by up to 50 million doses it had promised the EU. This threat was met with widespread criticism from international organizations and civil society, resulting in a swift U-turn by the EU. However, on March 5th, Italy and the EU did block 250,000 vaccines from leaving a factory in Rome destined for Australia. AstraZeneca blamed production problems at a plant in Belgium, but the EU said its contract had identified a number of manufacturing plants in the EU and UK that could supply the EU-bloc. The company recognized its contract with the EU, but noted that the EU-bloc could only receive vaccines from UK plants *after the UK had sufficient supplies*. The UK Government had insisted on a clause in its contract with AstraZeneca stating that the government “may terminate the deal and invoke what appear to be punishment clauses” if there was a delay in supply to the UK. The EU contract did not include such a clause [22]. More recently, the EU announced that it will use a plant in South Africa to produce the Johnson and Johnson vaccine, but those doses will be for import to the EU [23]. The African continent’s proportion of the population vaccinated remains very low, with many of its most vulnerable people yet to be vaccinated. Media reports cited a confidential contract between Johnson and Johnson and the South African government that prevented the country from imposing restrictions on vaccine exports, but the WHO also recognized that the arrangement was ‘presumably’ part of the company’s investment in vaccine hubs and manufacturing capacity in Africa.

Economic history tells us that export bans and trade embargoes are short-lived, ineffective policy tools; they are blunt instruments that damage vulnerable groups with doubtful political gains [24]. The hasty U-turn on vaccines suggests the EU questions the effectiveness of export bans, and the ban on exports to Australia is likely to achieve little for Italy. Indeed the most famous European trade embargo – Napoleon’s Continental System from 1806 to 13 – ultimately backfired. The system was intended to paralyze Britain through economic blockade by Europe and Russia. Instead, Europe suffered significant economic losses from the dislocation of international trade, with hyper-inflation, and huge damage to industrial progress [25]. In the next section, I suggest that the inequitable allocation of vaccines to middle- and low-income countries is doing significant damage to the entire global economy, in much the same way the Napoleon’s trade embargo wreaked havoc on the European economy.

### Intellectual property and the trade-related intellectual property rights (TRIPS) agreement

For the Covid-19 pandemic to end, or for endemic Covid-19 to become controllable, all countries will need urgent access to vaccines. Six independent human rights experts, under the auspices of UN ‘Special Procedures’, have written joint letters to G7 States, G20 States, the EU, the WTO, and pharmaceutical companies urging them to use all means possible to generate faster access to vaccines. Specifically, they have called for a temporary waiver of Covid-19 related intellectual property rights under the WTO Trade-Related Intellectual Property Rights (TRIPS Agreement) [26]. This follows calls for a TRIPS waiver from both India and South Africa [27].

The current TRIPS agreement allows for specific provisions for compulsory licensing and parallel importation that can be challenged by governments and manufacturers, but does not include an intellectual property waiver. However, there is precedent for a waiver in response to the widespread outbreak of a disease: a waiver was implemented as a result of the HIV/AIDS epidemic in Southern Africa in 2001, and there are other precedents for waivers in the area of intellectual property [28, 29].

The UN Committee on Economic, Social and Cultural Rights (CESCR) has affirmed that States parties must act on the ‘best available scientific evidence to protect public health’ and that ‘no one should be left behind’ [30]. The CESCR has recognized that ‘intellectual property regimes primarily protect business and corporate interests and investments’ and that ‘legal entities are included among the holders of intellectual property rights […] their entitlements, because of their different nature, are not protected at the level of human rights’. The International Covenant on Economic, Social and Cultural Rights (ICESCR), signed by 171 States, seeks to ensure non-discriminatory realization of economic, social and cultural rights [31]. States who are parties to this agreement can therefore be argued to have an obligation to grant all people access to Covid-19 vaccines, diagnostics, and therapeutics, based on the rights to life, health equality and science [32].

Furthermore, the UN has recognized that enormous State funding support was provided to pharmaceutical companies to supplement their own research and development drives for Covid-19 vaccines [33]. This State support built on a very large existing body of research that was available prior to the pandemic. The CESCR has stressed that private actors, including pharmaceutical companies ‘have the obligation, at a minimum, to respect Covenant rights […] including in relation to access to medicines and vaccines’, and that this extends to ‘medicines, comprising active pharmaceutical ingredients, diagnostic tools, vaccines, biopharmaceuticals and other related health-care technologies’, and should, therefore, ‘refrain from invoking intellectual property rights in a manner that is inconsistent with the right of every person to access a safe and effective vaccine against COVID-19 or the right of States to exercise the flexibilities of the TRIPS Agreement’ [34]. Medicines Sans Frontiers (MSF) has argued that EU regulations do not address legal barriers related to regulatory data and trade secrets, which are critical to the production of vaccines by alternative manufacturers, and the implementation of compulsory licenses by EU Member States [15]. The EU also does not have measures in place to ensure that pharmaceutical companies engage in initiatives to promote the global sharing of Covid-19 vaccine technologies. MSF has generated several key recommendations, including that the EU support the temporary intellectual property waiver for Covid-19 technologies at the WTO, and that the EU should not utilize trade policies and instruments to exert pressure on other countries so that they do not waive intellectual property rights.

### Global threats from inequitable access to Covid-19 vaccines

Global public goods tend to be under-provided and distributed in inequitable ways. Vaccine nationalism and export bans on Covid-19 vaccines by nation-states help to ensure that effective vaccination does not become a global public good. If Covid-19 vaccines, diagnostics and therapeutics are not rapidly and equitably provided to all global citizens, whether they live in high-, middle-, or low-income countries, significant global threats to health, security and prosperity will remain. The global threats from Coivd-19 affect all global citizens, not just those living in lower resource settings.

First, Covid-19 will continue to mutate, and may become more transmissible, more deadly, and resistant to vaccines. While there is a large pool of unvaccinated population in middle- and low-income countries, there is still a significant threat that vaccine-resistant Covid-19 mutations could emerge. Vaccine-resistant mutations will be an enormous threat to health and prosperity in all countries. The uneven roll out of vaccines and the emergence of the delta variant in India has already shown how mutations may become much more transmissible, and spread globally at a devastating rate. It is possible that we may become trapped in continuous cycle of new, vaccine resistant variants emerging with the need to continuously develop new vaccines. Immunizing the global population as quickly as possible is not just an altruistic gesture from the global North to the global South but an important element of self-interest.

Second, Covid-19 has produced a global economic crisis which will disproportionately affect middle- and low-income countries. Estimates of the economic impact of vaccine nationalism vary considerably, but all point to significant economic damage to the world economy, much of it falling on middle- and low-income countries. The RAND corporation has estimated that vaccine nationalism could result in a global loss of about USD $1·2 trillion per year, or 2·4% of GDP [35]. A more recent International Monetary Fund report has estimated that the new Covid-19 variants could result in a loss of USD $4.5 trillion by 2025, while a study commissioned by International Chamber of Commerce Research Foundation estimated that the global economy stands to lose as much as USD $9.2 trillion if governments fail to ensure developing economy access to COVID-19 vaccines [36, 37]. While the exact size of the global economic impact is under debate, it is clear that the next decade will see fragile economic growth, weak financial institutions, and astronomical national debts.

Third, Covid-19 is a threat to global security, which is also a global public good. Russia and China have been accused of using ‘vaccine diplomacy’ to expand their geopolitical spheres. Meanwhile, economic instability produces social instability, which can drive authoritarianism and conflict, and invites proxy conflicts in poorer countries by global powers. The longer the pandemic lasts and the worse the economic impact gets, the greater the chances are that conflict will occur within, or between, nation-states. The equitable allocation of vaccines to middle- and low-income countries should be seen as a complementary activity towards ensuring the prevention of conflict and promotion of global security.

Finally, Covid-19 is exacerbating entrenched health and economic inequalities. In particular, the health of the worst off in middle- and low-income countries will be most impacted by poor access to vaccines. Some authors have proposed that Covid-19 should be thought of as a syndemic rather than a pandemic [38]. A ‘syndemic approach’ examines the biological, social and environmental interactions that synergistically impact health [39]. Covid-19 is interacting with non-communicable diseases like hypertension, obesity, respiratory diseases and cancer, and with deeply entrenched social and economic inequalities. The syndemic nature of Covid-19 means that the negative health impacts of vaccine nationalism and export bans will be amplified for the worst off in middle- and low-income countries.

## A new way forward for global public goods

In the preceding sections I have made a case for Covid-19 vaccination to be considered a global public good, and argued for the equitable allocation of vaccines to middle- and low-income countries to help achieve that goal. COVAX and other elements of the global response to Covid-19 have shown some promising results, but national policies reflecting vaccine nationalism, splendid isolation, and trade embargos are hindering global progress. Covid-19 vaccines are being inequitably allocated to middle- and low-income settings, posing significant threats to global health and prosperity.

To address these threats we need stronger institutions for the provision of global public goods, and new ways of promoting legitimate and democratic international cooperation. Citizens of all nations are inextricably linked by Covid-19, but national politics has met its match. International relations are still dominated by national governments of sovereign nation-states, and we cannot rely on nation-states to always act in the interests of the global good. That said we have made significant steps forward in international collaboration in the last hundred years or so, establishing the UN, WHO, World Bank etc., and most recently COVAX during the pandemic. Covid-19 has provided a unique opportunity to broaden of the global discourse on the importance of justice and fairness for the successful governance, finance and delivery global public goods. Global governance is not a perfect science; it is a political process that encompasses both weak and strong actors.

We need a new vision for global democratic engagement in a world where global public goods are increasingly vital to all humanity; and, we need a system for improving the legitimacy and democratization of global policy-making. When national interests do not align with the global good, international institutions, including public-private and philanthropic organizations, step in. New public-private partnerships, such as the Global Fund to Fight Aids, Tuberculosis and Malaria and GAVI, have demonstrated impressive results. However, there are many potential conflicts of interest for public-private partnerships, and more importantly they are not democratically elected or publicly accountable.

Social psychologists have identified ten ‘basic personal values’ recognized in over 80 cultures: self-direction, stimulation, hedonism, achievement, power, security, conformity, tradition, benevolence and universalism [40]. Benevolence and universalism relate to self-transcendence or having concern for the well being of all. In many cultures people practice ‘strong reciprocity’: they tend to co-operate as long as others do too, but are ready to punish defectors and free riders even if it costs them personally [41]. We need to place peoples’ benevolence, universalism and reciprocity at the heart of governance for global public goods. It is quite possible that global citizens have strong preferences concerning the equitable allocation of Coivd-19 vaccines, but may lack an effective mechanism to express them. In this context, global citizenship reflects a notion that and individual’s national or local identity can be transcended, and that rights and responsibilities can be derived from belonging to humanity itself.

A number of Civil Society Organizations (CSOs) have responded to Covid-19 with calls to action on equitable vaccine access (note the list below is not intended to be comprehensive, it is only intended to provide some important examples of CSO responses). The People’s Vaccine campaign (a large coalition of international organizations and activists campaigning for free and fair for all Covid-19 vaccines) have suggested that ‘a vaccine paid for by the people should work for the people and remain of the people’ [42]. Public Citizen has called for an end to vaccine apartheid, suggesting the US alone could help produce the billions of doses needed to vaccinate everyone in lower resource settings. They suggest a $25 billion investment in the US vaccine manufacturing would be enough to accomplish this in one year [43].

Oxfam has called for the pandemic to be ended with a ‘people’s vaccine’. They too have criticized high-income countries for taking a ‘me first’ approach to vaccines by procuring more than 50% of the global supply, with severe consequences for low-income countries [44]. PrEP4All, an organization of community members, health care professionals and academics dedicated to improving access to HIV medications, is using its expertise to help expedite Covid-19 prevention research and ensure access to any Covid-19 vaccines, diagnostics and therapies are universally accessible [45]. The Health Global Access Project, has called on the US to commit to comprehensive Covid-19 technology transfer to expand manufacturing capacity in the global South, accompanied by greater transparency in Covid-19 related contracts between the government and the private sector [46]. Other CSOs that have been very active on Covid-19 vaccines include the Access Campaign (part of Médecins Sans Frontières), the People’s Health Movement Europe, the International Women’s Rights Action Watch Asia Pacific, and the Cairo Institute for Human Rights. COVAX has also selected CSO representatives to advise a number of its working groups.

However, CSOs assume many different organizational roles and forms, and have complex relationships with governments, international organizations, and public-private partnerships. CSOs like other types of organizations must form and refine their own culture, identity, and mission over time. In particular, it is possible that the missions of CSOs may evolve and change over time in ways that steer different CSOs in different directions (which could mean weaker alignment with public preferences).

I would argue that we need greater and more effective ways for public engagement on global public goods. Mobilizing the world’s marginalized populations could give voice to their experiences of being on the receiving end of inequitable policies, and help empower global institutions in providing global public goods. COVAX has stated that engaging with the public has been crucial in public health campaigns in recent years [47]. Moon and colleagues have argued that we need a global fund for global public goods coupled with a legitimate global political process to decide upon priorities [10]. The need for effective vaccination as a global public good will likely rise in the future, strengthening global arrangements for global public goods today will be a good investment in health for years to come. There is good evidence to show that a broad set of public constituents can be engaged in health policy-making, and strong arguments that public engagement while in a pandemic, and planning for future pandemics, is paramount  [48].

### Deliberative public engagement, Covid-19 vaccines, and global public goods

In this section I provide an example of one possible approach to more effective deliberative public engagement and global public goods. To do so, I construct a hypothetical example based on COVAX and Covid-19 vaccines. I have chosen COVAX purely as an illustrative example, this type of approach could also be incorporated into vaccine allocation decisions by national governments, the WHO, and non-profit organizations. This is not intended as a fully developed ‘business case’ for COVAX to consider, rather an illustrative example. Deliberative public engagement ‘events’ can be used to elicit the values and principles that civic-minded citizens from different countries feel ought to guide Covid-19 vaccine resource allocation decisions within and across high-, middle- and low-income countries. Findings from these events could be used to develop recommendations for policy-makers, either at the national level or for organizations with global reach, such as COVAX.

Health policy-makers are increasingly engaging the public to help solve complex policy problems, with deliberative public engagement emerging as a key approach [49]. This approach brings citizens together in a process of learning and dialogic exchange focused on collective problem-solving that is not consensus driven [28]. The example I describe below is based on the ‘mini-public’ model developed by Burgess and O’Doherty, which has been used in health policy decisions in Australia, Canada, the US and UK [50–52]. The approach involves bringing between 20 and 40 people together as a ‘mini-public’ to draw on citizens’ cultural and life experiences to identify important trade-offs and consequences of different policy choices, and the social values that underpin them [51]. It is well-suited to address ethically and politically challenging health policy decisions, and has been used for numerous policy topics including biobanks, human and animal genomics, data privacy, rare diseases, cancer, and technology assessment [50–52]. Another approach is deliberative polling, which focuses on bringing together large representative groups of people, with participants deliberating and voting on policy choices [53]. Empirical evidence shows that the public can make coherent and sophisticated recommendations concerning values and complex policy questions [54]. Public engagement can enhance accountability in institutional decision-making, improve the legitimacy of decisions, and help resolve moral conflict through shared decision-making [49, 50]. The ‘mini-public’ model could be a viable approach to improving participatory governance at a global level, with a strong emphasis on engaging publics from middle- and low-income countries on an ongoing basis.

The governance model for COVAX is complex to “ensure accountability and transparency, and diversity of geographic and thematic representation” [55]. The Office of the COVAX Facility supports operations, deal making and portfolio management, country engagement, finance and Advance Purchase Agreements. The COVAX Facility is overseen by the Board of Gavi. Additional elements of governance include the COVAX Shareholders Council, the Advance Market Commitment Engagement Group, and a range of technical and advisory working groups. A high level body called the COVAX Coordination Meeting coordinates efforts across all of the elements. But COVAX has only engaged ten representatives from CSOs to help different parts of the organization. A hypothetical ‘mini-public’ model for COVAX would consist of the following steps:

***1. Form a Steering Committee*** comprised of decision-makers, experts and key stakeholders to frame the issues and the possible ways to address them, including the development of agendas, potential deliberative questions, and supporting information for the deliberative public engagement events. In the COVAX example, the Steering Committee could be the Board of Gavi, but more likely would be drawn from the body that makes up COVAX Coordination Meeting. Strong representation from middle- and low-income countries on the Steering Committee is vital.

***2. Set the Deliberative Questions.*** The questions taken to the deliberative public engagement events should be developed in collaboration by the Steering Committee and other key stakeholders, including representatives from middle- and low-income countries. The questions will inform the supporting information for the deliberative events – in the form of ‘information briefs’ for participants (specifically designed booklets, web pages or videos in languages appropriate for different settings). The Office of the COVAX Facility could provide the scientific, clinical and social context for the policy topics.

For example, participants may be asked to deliberative over ethical principles and approaches for vaccine allocation, to identify policy recommendations, trade-offs, and principles upon which they agree or persistently disagree. Participants could address the two ethical approaches proposed by the WHO: 1. that countries initially receive vaccine doses proportional to population; or, 2. that vaccines are distributed to countries according to the number of frontline health care workers, the proportion of population over 65, and the number of people with co-morbidities in the country [56]. Participants could also deliberate on the three ethical approaches for fair vaccine allocation proposed by Emanuel and colleagues: 1. reducing premature deaths (giving priority to countries that would reduce more standard expected years of life lost (SEYLL) averted per dose of vaccine); 2. reducing serious economic and social deprivations (giving priority to countries that would reduce more poverty, avert more loss of Gross National Income, and avert more SEYLL per dose of vaccine); and, 3. returning to full functioning (giving priority to countries with higher transmission rates) [57].

***3. Recruit Participants.*** Each event should bring together a group of approximately 20–40 citizens recruited to reflect a diversity of life experiences and social perspectives from the general public of each target country or region. Participants should be stratified by age, gender, geography (e.g. urban/rural/remote) etc. Oversampling from historically marginalized groups is desirable (e.g. indigenous people, those living in poverty, remote populations) to ensure the voices of the most vulnerable are represented.

***4. Conduct the Deliberative Events.*** Typically each deliberative event takes place over two weekends led by trained facilitators. The first weekend set the foundations for meaningful deliberation, by providing participants with information on a range of perspectives related to global public goods and ethical approaches to vaccine allocation. This would include different perspectives from expert speakers, including patients, physicians, policy-makers, CSOs, indigenous representatives etc. The purpose of the second weekend is to enable participants to draw on their new skills and knowledge to make policy recommendations (e.g. on ethical frameworks for vaccine allocation). The events are intended to provide a forum to encourage all participants to deliberate by establishing deliberative norms, such as listening with respect, being open to others’ viewpoints, and seeking and providing clarity on positions. Face-to-face deliberations are preferred to online events, but are logistically more challenging. While it is possible to conduct deliberative events synchronously online, we have a lot of work to do to establish the quality of deliberation of online events compared to face-to-face. This is an important topic for future research.

***5. Qualitatively Analyze Data.*** Deliberative proceedings are not amenable to straightforward thematic or content analysis, since participants’ statements on the first and last day cannot be weighted equally, and more significance must be accorded to statements that are more informed and more collectively-oriented than other statements when characterizing the results of deliberation. Both deductive and inductive coding should be used. Deductive coding begins with established categories and codes, which are developed from the literature and research experience, and informed by the deliberative questions. Inevitably, coding deductively imposes constraints on what discursive content is captured. To combat these constraints, inductive coding methods should be used to capture emergent ideas.

***6. Develop Recommendations for Policy.*** After each event, the COVAX team should prepare a summary of the group recommendations, issues on which there were agreement or persistent disagreement, ratified by the event participants. Event summaries should be made publicly available. Event should be evaluated to: 1. better understand methods relating to how a deliberatively engaged public can best assess and provide advice on vaccine allocation decisions; and, 2. support and assess the ability of key decision makers to incorporate results of public engagement in decisions and policies. The Steering Committee would take these recommendations to COVAX as inputs for decision-making.

An often stated goal of deliberative public engagement is public empowerment to strengthen the conditions for autonomy and agency with an eye to sharing or redistributing power [58]. In this way, powerful interests such as national governments and pharmaceutical companies would hold less power in policy debates and ethical discussions, with greater power given to different publics. Deliberative public engagement could be used with COVAX to empower different publics in COVAX vaccine allocation decisions. At present, COVAX only includes 10 community and civil society representatives: much greater and more effective engagement is needed. If deliberative public engagement is also employed with national government decision-makers in their vaccine allocation decisions, it could empower the public in different countries to change their governments’ policies, reducing vaccine nationalism. This might take the form of pressuring national governments to make greater donations to COVAX, lobbying governments for a TRIPS waiver, encouraging governments to strengthen multilateral agreements on vaccines (rather than weakening them), or pushing pharmaceutical companies to establish more manufacturing hubs in low-income countries (producing vaccines for those countries). A commitment to public engagement should be underpinned by two principles [58]: 1. It is unwise or dangerous to place too much power in too few hands; and, 2. Shared power is essential for achieving a more balanced understanding of the various scientific, economic, societal and global consequences of policy choices relating to global public goods and Covid-19 vaccines. In doing so, public engagement can help ensure that global citizens from across the planet could be given the opportunity to contribute to policy debate and decision-making. All too often politicians and decision-makers fail to appreciate public values and sentiment. If different publics believe in the global equitable allocation of Covid-19 vaccines, empowering them is critical to changing policies based on vaccine nationalism.

## Conclusion

Covid-19 presents a unique opportunity to transform democratic engagement in the governance of global public goods. Systematic approaches to engaging publics, especially from middle- and low-income countries, have yet to be fully developed for international organizations involved in providing global public goods. Improving the representation of publics from middle- and low-income countries could go a long way to addressing concerns about the equitable allocation of vaccines. It is difficult to overstate the importance of global public goods in the twenty-first Century: we face existential threats from the climate crisis and biodiversity loss (clean air, habitable temperatures, and biodiversity are all global public goods). Global public goods are central to preparing for future pandemics, as well as UN Sustainable Development Goals on sustainability, climate change, and biological diversity. To make us more resistant and resilient to future global health crises we need transformative thinking to democratically engage global citizens. We need to lay the foundations for a ‘global social contract’ on global public goods.

## Data Availability

Data sharing not applicable to this article as no datasets were generated or analysed during the current study.

## Abbreviations

ACT: Access to Covid-19 tools accelerator
CEPI: Coalition for epidemic preparedness innovations
CESCR: Committee on economic, social and cultural rights
COMEST: Commission on the ethics of scientific knowledge and technology
CSO: Civil society organization
C-TAP: Covid-19 technology access pool
GAVI: The vaccine alliance
GFPG: Global fund for public goods
IBC: International bioethics committee
ICESCR: International covenant on economic, social and cultural rights
IMF: International monetary fund
MSF: Medicines sans frontiers
SEYLL: Standard expected years of life lost
TRIPS: Trade-related intellectual property rights
UN: United Nations
WHO: World Health Organization
WTO: World Trade Organization
